# Bidirectional association between polycystic ovary syndrome and periodontal diseases

**DOI:** 10.3389/fendo.2023.1008675

**Published:** 2023-01-23

**Authors:** Yang Dou, Jinglei Xin, Peng Zhou, Jianming Tang, Hongliang Xie, Wanting Fan, Zheng Zhang, Donglei Wu

**Affiliations:** ^1^ Department of Stomatology, Shenzhen Baoan Women’s and Children’s Hospital, Jinan University, Shenzhen, Guangdong, China; ^2^ Department of Stomatology, Guangdong Women and Children hospital, Guangzhou, Guangdong, China; ^3^ Department of Stomatology, Shenzhen People’s Hospital, Shenzhen, Guangdong, China

**Keywords:** polycystic ovary syndrome, periodontal diseases, host immune, inflammation, oral micro biota

## Abstract

Polycystic ovary syndrome (PCOS) and periodontal disease (PDD) share common risk factors. The bidirectional interaction between PCOS and PDD has been reported, but until now, the underlying molecular mechanisms remain unclear. Endocrine disorders including hyperandrogenism (HA) and insulin resistance (IR) in PCOS disturb the oral microbial composition and increase the abundance of periodontal pathogens. Additionally, PCOS has a detrimental effect on the periodontal supportive tissues, including gingiva, periodontal ligament, and alveolar bone. Systemic low-grade inflammation status, especially obesity, persistent immune imbalance, and oxidative stress induced by PCOS exacerbate the progression of PDD. Simultaneously, PDD might increase the risk of PCOS through disturbing the gut microbiota composition and inducing low-grade inflammation and oxidative stress. In addition, genetic or epigenetic predisposition and lower socioeconomic status are the common risk factors for both diseases. In this review, we will present the latest evidence of the bidirectional association between PCOS and PDD from epidemiological, mechanistic, and interventional studies. A deep understanding on their bidirectional association will be beneficial to provide novel strategies for the treatment of PCOS and PDD.

## 1 Introduction

Polycystic ovary syndrome (PCOS), the most common heterogeneous disorder in women with high prevalence rate of 5%–10% (1), is characterized by hyperandrogenism (HA), oligomenorrhea or amenorrhea, polycystic ovary, and low-grade inflammation (2). PCOS contributes to menstrual cycle abnormalities, pregnancy complications, long-term metabolic disorders, cardiovascular diseases, and even increases cancer risk (2). Periodontal diseases (PDDs) are multifactorial, highly prevalent chronic inflammatory disorders in the oral cavity. It is widely recognized that the symbiotic relationship between oral microbiota and the host is essential for the homeostasis in oral microecology (3). Oral flora dysbiosis renders dominance of periodontal pathogens, impacts tooth-supporting tissues (gingiva, periodontal ligament, and alveolar bone), and even leads to tooth loss (4). Systemic diseases including diabetes mellitus (DM), obesity, and metabolic syndrome (MS) are independent risk factors for PDD (5). Interestingly, PCOS and PDD have common risk factors, including metabolic syndrome (6), obesity, DM, and cardiovascular disease (7). Recently, several studies have explored and confirmed the association between PCOS and PDD (8–11). However, the cause-and-effect relationship between PCOS and PDD and their molecular mechanisms remain undefined. In this review, we will focus on recent studies to discuss the potential mechanisms between PCOS and PDD.

## 2 Epidemiological evidence of the bidirectional relationship between PCOS and PDD

There are 15 published papers on the bidirectional relationship between PCOS and PDD, including 14 cross-sectional studies and 1 randomized controlled trial. A recent cross-sectional study indicated that women who are infertile, in comparison with those who are fertile, are more vulnerable to PDD with signs of increased probing depth (PD) and clinical attachment loss (CAL). However, no difference were observed in oral-health-related quality of life (OHRQoL) scores between the two groups, which might reveal a poor periodontal status and weak awareness of oral health care in infertile women (12). PCOS as an important cause of infertility might be associated with PDD. Recently published studies have provided evidence to support the interaction network between PCOS and PDD (9–11). PCOS may have an impact on gingival inflammation and *vice versa*. Several cross-sectional studies unanimously concluded that PCOS increases the risk of PDD (13–18). Women with PCOS tend to manifest poor periodontal conditions, such as positive bleeding on probing (BOP), deep PD, and high plaque index (PI). Interestingly, controlling PCOS with oral contraceptives and metformin mitigates periodontal inflammation (19). A retrospective cohort study involving 48,820 subjects showed that PDD multiplies the risk of PCOS (20). The bidirectional relationship between PCOS and PDD was discussed in recent meta-analysis research. The risk of PDD is increased by 28% in women with PCOS, and the risk of PCOS is increased by 46% in women with PDD (9). In addition, a higher PD rate is observed in PCOS patients than in healthy women in a recent case–control study. However, there was no differentiation between PCOS and healthy women in other periodontal parameters, such as PI, BOP, and CAL (21). Although no causal relationship between genetic liability for PCOS and periodontitis was identified based on a recent bidirectional Mendelian randomization analysis (22), strong evidence supports the mutual effect between PCOS and PDD. The potential mechanisms between PCOS and PDD are discussed in the following sections (**Table 1**).

**Table 1:** Summary of studies investigating the association between PCOS and PDD.

Authors Year	Study style	Periodontal Index	Other Index	Sample	Group	Inclusion criteria of PD	Inclusion criteria of PCOS	Confounders variables assessed
Akcali (2014) (23)	Case-control	PI, BOP, PPD	/	/	PCOS+HP:45 PCOS+GG:35 NP+GG: 20 NP+HP: 25	BOP >50% sites, PPD< 3mm at 90% sites, no sign of PD	Rotterdam criteria (2003)	hyperandrogenism, DM, hyperprolactemia, congenital adrenal hyperplasia, thyroid disorders, Cushing’s syndrome, HBP, CVD, hepatic or renal dysfunction, oral contraceptives, steroid hormones, insulin-sensitizing. BMI>30 kg/m^2^;
Rahiminejad (2015) (17)	Case-control	PI, BOP, CAL,	/	/	PCOS:98 HC:98	/	Rotterdam criteria (2003)	Pregnancy, smoking, malignancies, osteoporosis, antibiotics, periodontal treatment, BMI >25 kg/m^2^, IGT
Işık (2020) (21)	Case-control	BOP, PI, GI, CAL,PD	/	/	PCOS:116 HC:90	/	Rotterdam criteria (2003)	Androgen-secreting tumors, congenital adrenal hyperplasia, Cushing’s syndrome, hyperprolactinemia, thyroid disorders, hypertension, hepatic or renal dysfunction, CAM, DM, chronic inflammatory disease, malignancy, pregnancy, AIDS, smoking, alcohol drinking, periodontal treatment, oral contraceptive agents, antipsychotic, antiepileptic, steroid hormones, antihypertensive, insulin sensitizing drugs, antibiotics, or anti-inflammatory drugs
Ozcaka (2012) (16)	Case–control	PPD, BOP, PI	IL-6	GCF, saliva, serum	PCOS+HP:30 PCOS+GG:31 HC:12	BOP >50% sites, PPD <3 mm at 90% sites, no sign of PD, >20 teeth	Rotterdam criteria (2003)	BMI > 30 kg/m^2^, androgen-secreting tumors, congenital adrenal hyperplasia, thyroid disorders, DM, hyperprolactinemia, Cushing´s syndrome, hypertension, hepatic and renal dysfunction, oral contraceptives, steroid hormones, insulin-sensitizing drugs, alcohol, smokers
Ozcaka (2013) (24)	Case–control	PPD, BOP, PI	IL-17	GCF, saliva, serum	PCOS+HP:30 PCOS+GG:31 HC:12	BOP >50% sites, PPD< 3mm at 90% sites, no sign of PD, >20 teeth	Rotterdam criteria (2003)	BMI > 30 kg/m^2^, hyperandrogenism, thyroid disorders, hyperprolactinemia, CVD,DM, high BP, oral contraceptives, steroid hormone, insulin-sensitizing drugs
Akcali (2015) (13)	Case–control	PI, BOP, PPD	MMP-8, TIMP1	Saliva, serum	PCOS+HP:45 PCOS+GG:35 NP+GG: 20 NP+HP: 25	BOP >50% sites, PPD <3 mm at 90% sites, no sign of PD	Rotterdam criteria (2003)	Hyperandrogenism, DM, hyperprolactemia, congenital adrenal hyperplasia, thyroid disorders, Cushing’s syndrome, HBP, CVD, hepatic or renal dysfunction, oral contraceptives, steroid hormones, insulin-sensitizing. BMI >30 kg/m^2^
Akcali (2017) (25)	Case–control	PI, BOP, PPD	MMP-8, TIMP1, MPO, NE	Saliva, serum	PCOS+HP:45 PCOS+GG:35 NP+GG: 20 NP+HP: 25	BOP >50% sites, PPD <3 mm at 90% sites, no sign of PD	Rotterdam criteria (2003)	Hyperandrogenism, DM, hyperprolactemia, congenital adrenal hyperplasia, thyroid disorders, Cushing’s syndrome, HBP, CVD, hepatic or renal dysfunction, oral contraceptives, steroid hormones, insulin-sensitizing. BMI>30 kg/m^2^
Dursun (2011) (15)	Cross-section	PPD, CAL, BOP, GI	MPO, NO	GCF	PCOS:25 HC:27	/	Rotterdam criteria (2003) (26)	Cushing syndrome, congenital adrenal hyperplasia, hyperprolactinemia, thyroid dysfunction, and androgen-secreting tumors, smokers, oral contraceptives, BMI>30 kg/m^2^; IGT
Saglam (2018) (27)	Case–control	BOP, PI, GI PPD, CAL	MDA, 8-OHdG, TAS	Serum, saliva	PCOS:22 PCOS+CP:22 NP+CP:22 NP+HP:22	PPD ≥5 mm, CAL ≥ 6 mm	Rotterdam criteria (2003)	BMI > 25 kg/m^2^, HbA1c >6,5%, OGTT-2h > 200 mg/dl, antibiotics, oral contraceptives, steroid hormones, hypertensive medications, insulin-sensitizing drugs, periodontal treatment, androgen-secreting tumors, congenital adrenal hyperplasia, thyroid disorders, DM, hyperprolactinemia, Cushing’s syndrome
Varadan (2019) (18)	Case–control	BOP, PPD, PI, mGI	MDA, MPO	Serum, saliva	PCOS:30 HC:30	/	Rotterdam criteria (2003)	Cushing’s syndrome, adrenal hyperplasia, hyperprolactinemia, thyroid dysfunction, androgen secreting tumors, history of systemic disease, pregnancy, interfering drugs (antibiotics, oral contraceptives, chemotherapeutic), periodontal treatment, smoking, tobacco, and alcohol consumption
Dharuman (2022) (28)	Cross-section	BOP, PD, PISA	AOPP	Serum; saliva	PCOS:12 CP:12 PCOS+CP:12 HC:12	2012 definition (29)	Rotterdam criteria (2003)	Smoking, pregnant, history of systemic disease other than PCOS, consumed medications within the past 3 months, and had periodontal therapy.
Saljoughi (2020) (30)	/	BOP, CAL, PD, raidograph	Visfatin	GCF	PCOS+HP:25 NP+CP: 23 PCOS+CP:30 NP+HP: 32	> 35 years and > 30% sites with CAL ≥3 mm, and PPD ≥5 mm with BOP (+).	Rotterdam criteria (2003)	Pregnancy, interfering drugs (antibiotics, oral contraceptives, antihypertensive, DM drugs), infection, thyroid disorders, hyperprolactinemia, diabetes, hypertension, malignancies, osteoporosis, obesity, overweight, smoking, alcohol consumption.
Zia (2022) (31)	Cross-section	PD, CAL, PI, GI	ALP, BMD, CTX, VD	Serum	PCOS:40 CP:40 PCOS+CP:40 HC:20	2017 classification (32)	Rotterdam criteria (2003)	BMI, hypo- or hyperthyroidism, hypogonadism, Cushing’s syndrome, androgen-secreting tumors, smoking, drugs-like oral contraceptives, anti-inflammatory, antibiotics, and others that could affect periodontal status in the last 6 months
Deepti (2017) (33)	RCT	PI, GI, BOP, CAL, PD	hsCRP HOMA	serum	SPR+MI:30 OHI+MI:30	≥20 teeth; moderate periodontitis (34)	Androgen Excess Society/2006 criteria (35)	androgen-secreting tumors, congenital adrenal hyperplasia, and thyroid dysfunction; nephrotic syndrome; chronic renal failure; significant cardiovascular disease; DM; active cancer within the last 5 years; smokers and women who are alcohol dependent; history of systemic antibiotics or oral contraceptives usage within last 3 months; periapical pathology or other oral inflammatory conditions; and any periodontal treatment within 6 months prior to study
Porwal (2014) (19)	Case-control	BOP, PPD, CAL, GI	hsCRP	Serum	PCOS:41 PCOS+T: 45 HC:40	/	Rotterdam criteria (2003)	BMI > 30 kg/m^2^, thyroid disorders, hyperprolactinemia, androgen-secreting tumors, chronic inflammatory diseases, DM, CVD, cancer, smoking, alcohol, antibiotics, periodontal treatment, aggressive periodontitis

AIDS, acquired immunodeficiency syndrome; ALP, alkaline phosphatase; advanced oxidative protein products, AOPP; BMD, bone mineral density; BMI, body mass index; BOP, bleeding on probing; CAL, clinical attachment loss; CTX, C-terminal telopeptides of type I collagen; CP, chronic periodontitis GCF, gingival crevicular fluid; GI, gingival index; GG, gingivitis; HC, healthy control women; hsCRP, high-sensitivity C-reactive protein; IL, interleukin; IGT, impaired glucose tolerance; MDA, malondialdehyde; MMP, matrix metalloproteinase; MPO, myeloperoxidase; NA, not applicable; NE, neutrophil elastase; NP, non-polycystic ovary syndrome; NO, nitric oxide; PCOS, polycystic ovary syndrome; PCOS+T, PCOS treatment; PI, plaque index; PISA, periodontal inflamed surface area; PPD, periodontal probing depth; RCT, randomized controlled trial; TAS, total antioxidant status; TIMP, tissue inhibitors of MMP-I; TNF-α, tumor necrosis factor-α, VD, 25-hydroxyvitamin D; /, no available.

## 3 Potential mechanisms by which PCOS might increase the risk of PDD

**Figure 1:**
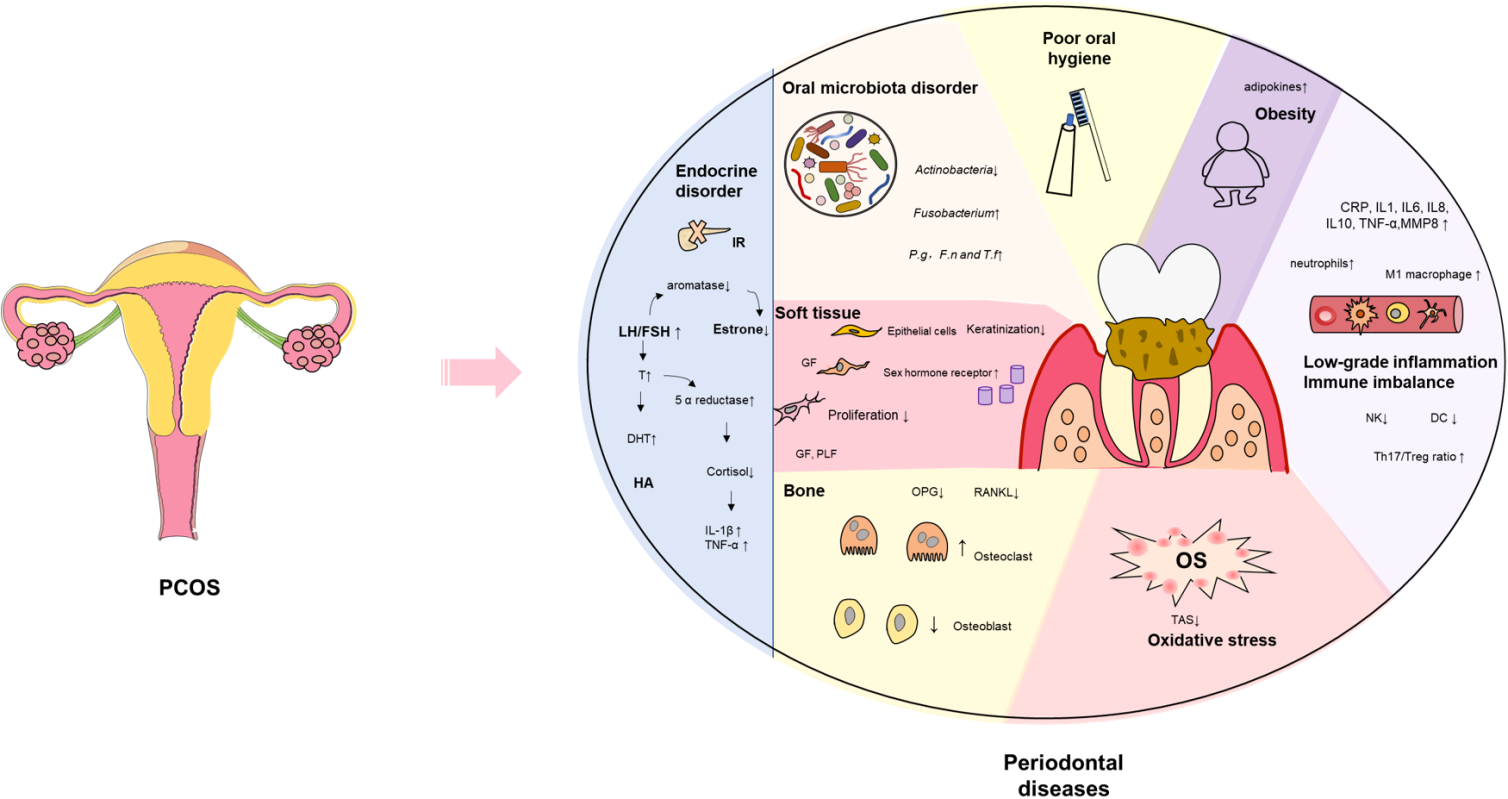
Schematic representation of the association between PCOS and PDD. PCOS is characterized by low-grade inflammation, immune imbalance, endocrine disorder, and oxidative stress. The above microenvironment has an adverse implication for oral microbiome, periodontium, and alveolar bone. In addition, PCOS women show a propensity for obesity and have a poor oral hygiene, which increases the risk of PDD. PCOS, polycystic ovary syndrome; T, testosterone; DHT, Dihydro testosterone; HA, hyperandrogenism; IR, insulin resistance; LH/FSH, luteinizing hormone/follicle stimulating hormone; *F.n*, *Fusobacterium nucleatum*; *P.g*, *Porphyromonas gingivalis*; *T.f*, *Tannerella forsythia;* GF, gingival fibroblasts; PLF, periodontal ligament fibroblasts; OPG, osteoprotegerin; RANKL, receptor activator of NF-κB ligand; MDA, malondialdehyde; MPO, myeloperoxidase; No, nitric oxide; TAS, total antioxidant status; NK, natural killer cells; DC, dendritic cell;IL, interleukin; TNF, tumor necrosis factor.

PCOS is featured with endocrine disorders, including HA, insulin resistance (IR), and estrogen reduction. Endocrine disorder aggravates the development of PDD. We summarize the potential mechanisms of endocrine disorders between PCOS and PDD in the following four aspects (**Figure 1**).

### 3.1 PCOS alters the composition of oral microbiota

The initiation and progression of periodontal lesions depend on the interactions between host and oral microecology. Within the oral ecosystem, local microbial metabolism, systemic stress-induced dysbiosis, especially hormonal imbalance, toxins, and inflammatory cytokines might result in 10-fold higher migration of immune cells from the gingival sulcus (36). Once the host immune system is continuously activated, chronic inflammation will develop. Periodontitis is mainly caused by the disturbance of subgingival biofilm ecosystems. *Fusobacterium nucleatum* (*F.n*) is one of the anaerobic bacteria in supra- and subgingival biofilms. *F.n* serves as a bridge to assist the interspecies coaggregation, including *Porphyromonas gingivalis* (*P.g*), *Treponema denticola* (*T.d*), and *Streptococcus gordonii* (*S.g*), and accelerates biofilm formation *via* outer membrane proteins. In addition, *F.n* breaks the epithelial barrier and engages in attacking the lymphocytes, resulting in the host immune imbalance (37). Accumulating evidence suggests that the alteration of the oral microbial community in PCOS women might increase the risk of PDD. Increased number of *Fusobacterium* and decreased number of Actinobacteria were observed in the salivary microbiome from PCOS women (38). Actinobacteria are more abundant in the periodontium in a healthy state than that in the inflammatory state (39) and plays an important role in maintaining oral microbial homeostasis. A decreased proportion of phylum Actinobacteria was observed in the salivary microbiome in PCOS when compared with healthy women, but no relationship was found between saliva alpha diversity or beta diversity and serum testosterone and inflammatory markers in PCOS (40, 41). Higher abundances of *F.n* and *Tannerella forsythia* (*T.f*) were observed in gingival crevicular fluid (GCF) in patients with comorbidity of PCOS and gingivitis rather than patients with gingivitis alone (23), which suggests a detrimental effect of PCOS on periodontal microecology (**Table 2**). Interestingly, a similar change was observed in the abundance of *F.n* and Actinobacteria in DM patients accompanied with PDD (43).

**Table 2:** Alteration of composition of oral microbial community in PCOS women.

Case group	Control group	Sample types	Methods	Result*
Adolescent PCOS (42)	Health	GCF	qPCR	*P.m* and *T.d*↓
PCOS (40)	Health	Saliva	16S rRNA	Actinobacteria↓
PCOS (38)	Health	Saliva	16S rRNA	Fusobacterium↑Actinobacteria↓
PCOS and gingivitis (23)	Gingivitis	Saliva	qPCR	*P.g*, *F.n* and *T.f*↑

GCF, gingival crevicular fluid; F.n, Fusobacterium nucleatum; PCOS, polycystic ovary syndrome; P.g, Porphyromonas gingivalis; P.m, Peptostreptococcus micros; qPCR, quantitative real-time polymerase chain reaction; T.d, Treponema denticola; T.f, Tannerella forsythia; *the trend for compared with control group. ↑, increased level; ↓, decreased level.

Sex hormones may also contribute to the differences in the oral microbiota. A higher abundance of *F.n* subspecies, *fusiforme*/*vincentii*, was observed in women compared with men (44). Additionally, increased abundance of Actinobacteria has been reported in postmenarcheal girls compared with premenarcheal girls (45). Moreover, endocrine disorder alters the microbiota composition in female individuals at reproductive age. The level of estradiol (E2) was positively correlated with the number of green complex bacteria (*Capnocytophaga gingivalis*) in subgingival microflora in adolescent girls with PCOS (42). Similarly, alteration of the composition of oral microbial community was observed in various endocrine system diseases, such as DM and obesity (41). A recent study demonstrated that diabetes increased the pathogenicity of the oral microbiota by enhancing the expression of interleukin (IL)-17 in mice. Moreover, mice infected with DM-related oral microbiota manifest inflammation and bone loss in periodontal tissues (46), which confirms that oral microbiota is implicated in DM patients. In addition, altered composition of salivary bacteria is also observed in overweight women (47).

Both HA and IR affect the composition of the microbiota and metabolic activity in PCOS. HA was associated with decreased alpha diversity and alteration of specific Bacteroidetes and Firmicutes in gut microbiota in women with PCOS (48). PCOS accompanied by IR alters the composition of the gut microbial community (48) and oral microbiota. Numerous observational and interventional studies have linked IR to PDD (49). IR was positively correlated with the abundances of *Granulicatella*, *Veillonella*, *Streptococcus*, and *Scardovia* in supragingival plaque by 16s rDNA sequencing in patients with metabolism-associated fatty liver disease (50). A previous study has demonstrated that IR was associated with the abundance of 22 individual taxa in human subgingival plaque (51). Hence, endocrine disorder in PCOS might increase the risk of PDD by altering the composition of oral microbiota.

Microbial metabolomics is the key to probe into the relationship between the alteration of oral microbial composition in PCOS and the occurrence of PDD. Altered composition of salivary bacteria in PCOS women causes changes in host metabolism (38), including oxidative phosphorylation, methane metabolism, nitrogen metabolism, butanoate metabolism, molecular chaperones, folding catalysts, and membrane and intracellular structural molecules. More importantly, consistently upregulated methane metabolism and downregulated chaperones and folding catalysts are observed in PCOS (38). Interestingly, both butanoate and methane metabolism in the subgingival microbiota are significantly over-activated in patients with periodontitis (52). Butanoate production has been known to play an important role in periodontal disease (53). Butyrate, the metabolite of periodontal pathogens (including *F.n* and *P.g*) *(* 54), inhibits cell cycle of gingival fibroblasts and promotes apoptosis (55) by inducing reactive oxygen species (56). Therefore, more studies should focus on the effects of the oral microbiome in PCOS on host metabolism.

### 3.2 PCOS promotes bone resorption

PCOS has a negative effect on bone metabolism. PCOS women are at high risk of osteoporosis (57). Another study revealed a decreased bone mineral density (BMD) in the spine and femur and less bone formation with decreased osteocalcin in PCOS patients with a body mass index (BMI) <27 kg/m^2^ (58). Zia et al. evaluated the serum levels of bone metabolism and bone turnover markers (BTMs) in PCOS accompanied with PDD. Increased level of C-terminal telopeptides of type I collagen (CTX, bone resorption marker) and decreased level of alkaline phosphatase (ALP, bone formation marker) were found in patients with comorbidity of PCOS and PDD than in patients with PDD alone (31), suggesting that PCOS worsens the bone metabolism of the alveolar bone around the periodontal tissue. In addition, PCOS enhances the levels of PD and CAL in PDD patients with continuing alveolar bone resorption (31, 33).

HA, estrogen reduction, and IR are responsible for the alteration of bone metabolism in PCOS (57). Elevated luteinizing hormone (LH)/follicle stimulating hormone (FSH) ratio in PCOS decreases the activity of aromatase, which converts androgens into estrogens, leading to hyperandrogenism (57). Although androgen plays an important role in maintaining BMD in men, it does not exert the same function in women with PCOS (57). Excessive androgen has an adverse effect on bone anabolism in women. The increase in 5α reductase in PCOS enhances the conversion of testosterone to dihydrotestosterone (DHT), which inhibits the expression of cortisol. Subsequently, cortisol inactivation increases the expression of IL-1β and tumor necrosis factor alpha (TNF-α) and contributes to bone resorption. In addition, estrogen reduction in PCOS decreases bone density, deteriorates microarchitecture, and increases fracture risk (59). IR in PCOS women inhibits the expression of osteoprotegerin (OPG) and induces RANKL expression, which are responsible for bone resorption (57). Decreased levels of vitamin D and increased levels of parathyroid hormone (PTH) and calcitonin in PCOS promote bone resorption (57). These findings suggest that hormonal imbalance in PCOS might increase bone resorption and lead to the development of PDD.

### 3.3 PCOS increases infection susceptibility in soft tissues

Poor oral hygiene and the inflammation status of gingival tissues are closely related to periodontitis. Higher PI and gingival index (GI) are observed in PCOS patients than in healthy controls (31), which indicates poor oral hygiene and soft tissue inflammation in PCOS patients. Accumulated evidence demonstrated that periodontal tissues are hormone sensitive and that gonadal hormones modulate the periodontium including fibroblasts and the epithelium (60, 61). Hence, endocrine dysfunction in PCOS might impact the periodontium. The tissue specificity of sex steroid hormone mainly depends on the expression of specific hormone receptors (62). Extensive studies have supported the high expression of estrogen receptors in periodontal tissues. Estrogen receptors (ERs), but not progesterone, are highly expressed in gingival tissues of PCOS (63). During inflammation, estrogen receptors are expressed in gingival tissues by 10-fold than normal state (64). Meanwhile, androgen receptors have been detected in the nuclei of basal gingival epithelial cells and gingival fibroblasts (65). Collectively, androgens and estrogens are preferentially localized and retained in periodontal tissues (66). Moreover, inflammation increases the metabolism activity of androgens in gingival tissues (67–69). Testosterone can be metabolized to 5α-dihydrotestosterone, 4α-androstenedione, and 5α-androstanediols in human gingival fibroblasts *in vitro (* 70). In addition, sex hormones also mediate the action of periodontal ligament fibroblasts, gingival fibroblasts, and epithelial cells in the gingiva. Estrogen plays an important role in maintaining the epithelial barrier in the periodontium (60). It has been reported that estrogen stimulates the proliferation and keratinization of gingival epithelium, increases the downgrowth of epithelial attachment, and accelerates the proliferation of fibroblasts (62). Decreased estrogen contributed to the thinning of oral mucosa through reducing the epithelial keratinization and collagen formation in connective tissues (71, 72). Progesterone, however, is not conducive to the repair and maintenance of the periodontium (60). Progesterone inhibited the proliferation of human gingival fibroblasts *ex vivo (* 73). In addition, progesterone suppressed the collagen synthesis in periodontal ligament fibroblast (74). In summary, androgens might modulate the gingival tissues in PCOS by affinity for androgen receptors, elevation of androgen metabolism, and inhibition of fibroblast proliferation.

### 3.4 PCOS causes immune imbalance

Immune–endocrine interactions play an important role between PCOS and PDD. However, no study has directly explored the effects of PCOS-induced immune dysfunction on the development of PDD. PCOS-induced innate and adaptive immune imbalance might promote the pathogenic effects by periodontal pathogens. High levels of neutrophils and high ratio of neutrophil-to-lymphocyte in PCOS indicate low-grade inflammation status (75, 76). In addition, a shift from M2- to M1-polarized macrophage was observed in a dehydroepiandrosterone-induced mouse model of PCOS, which caused chronic inflammation (77). The increased M1/M2 ratio accelerated alveolar bone resorption in periodontal tissues (78). In addition, decreased natural killer (NK) cells and dendritic cells (DC) have been reported in PCOS women (79), which might enhance susceptibility to periodontitis (80). T lymphocytes play an important role in adaptive immune response, while the dysfunction of T lymphocytes might accelerate microorganism invasion. PCOS patients are characterized by Th1/Th2 imbalance and increase in CD4^+^CD28^−^ T cell and Th17/Treg ratio (79).

Sex hormones have been shown to influence the immune system in the periodontium. Increased immune cells were observed in oral and sulcular gingival epithelium during pregnancy (81). Androgens play an important role in adaptive immunity and innate inflammatory response and maintain the homeostasis of periodontal tissues (82). Furthermore, sex hormone disorders in PCOS have been shown to mediate the production of cytokines (62). E2 level was negatively correlated with interferon gamma (IFN-γ) level and positively correlated with PD-1 level in serum CD4+ and CD8+ T cells of infertile women with PCOS (83). This immune network is complicated and can be an interesting topic for future research.

### 3.5 PCOS elevates the systemic inflammation

PCOS is characterized by low-grade chronic inflammation, which is considered as a key contributor to the development of PDD. In fact, systemic inflammation in metabolic diseases creates a chronic inflammatory status for periodontal tissues (84). A large number of studies revealed that multiple inflammatory cytokines contributed to the interaction between PCOS and PDD, including IL-6, IL-17, and TNF-α. Reduced estrogen in PCOS increases the expression of proinflammatory cytokines, including IL-1, IL-6, IL-8, IL-10, TNF-α, and granulocyte colony-stimulating factor (GCSF), which creates an inflammatory microenvironment for the development of PDD (71, 72). In addition, a higher expression of matrix metalloproteinase (MMP)-8 was observed in the serum and saliva of women with comorbidity of PCOS and gingivitis compared with that in systemically healthy individuals with gingivitis (13). A positive correlation between MMP-8 levels and poor periodontal conditions including PD, BOP, and PI was detected in PCOS, which suggests a deteriorative role of PCOS for PDD (13). Similarly, elevated serum CRP level in PCOS reveals systemic inflammation and increases the risk of PDD (19, 33).

### 3.6 PCOS triggers oxidative stress

The imbalance of oxidation/antioxidant capacity contributes to oxidative damage, called oxidative stress (OS). The PCOS-induced OS has an impact on gingival inflammation. A higher level of malondialdehyde (MDA) (a lipid peroxidation product indicating OS) in serum and GCF was detected in women with PCOS, which was positively correlated with gingival inflammation (18, 27). In addition, the contents of nitric oxide (NO) and myeloperoxidase (MPO) were higher in women with PCOS than in healthy women (14). Furthermore, women with PCOS and PDD exhibited higher serum levels of 8-hydroxy-2′-deoxyguanosine (8-OHdG), MPO, MDA, and lower total antioxidant status (TAS) than women with PCOS alone (25, 27). Hence, PCOS might enhance systemic lipid peroxidation and oxidative DNA damage and contribute to the development of PDD.

### 3.7 PCOS with obesity increases the expression of inflammation mediators

Epidemiological data showed that obesity affects 30%–70% PCOS women (85). Although not all morbidly obese women develop PCOS, adipose tissue is important for the development and maintenance of PCOS (85, 86). Adipokines including adiponectin leptin, resistin, visfatin, and retinol-binding protein 4 (RBP4) are mainly secreted by adipose tissues and involved in the glucolipid metabolism and IR (87). Disturbed expression of adipokines has been found in PCOS, which has an impact on the secretion of sex steroid, such as IR (87). A recent study identified a significantly elevated level of visfatin in GCF of women with comorbidity of PCOS and PDD, compared with that of women with PDD alone (30). In addition, more than 50 cytokines and inflammatory mediators are released from adipose tissues and responsible for the regulation of inflammation, glucose metabolism, and energy balance. Increased levels of CRP, IL-6, and TNF-α were detected in PCOS, which contributes to low-grade chronic inflammation and increases the risk of PDD (87). In summary, obesity in PCOS results in IR and low-grade chronic inflammation and accelerates PDD development through altering the expression of adipokines and inflammation mediators.

### 3.8 PCOS therapy mitigates periodontal inflammation

Porwal et al. (19) compared the morbidity in two groups of patients with PCOS receiving and not receiving drug treatment. A lower frequency of moderate periodontitis was observed in PCOS with the drug treatment group. Furthermore, the authors also evaluated the effect of drug treatment on periodontal clinical parameters and serum hsCRP levels in PCOS. Periodontal parameters including BOP, PD, and CAL were significantly improved by drug treatment. In addition, drug treatment caused lower serum level of hsCRP in PCOS. Interestingly, there were no statistical difference in the serum level of hsCRP between PCOS patients receiving drug treatment and healthy controls. The authors concluded that PCOS-induced systemic inflammatory responses might function as a pivotal role in the development of PDD. However, further studies are needed to focus on the impacts of drug therapy for PCOS on periodontal status based on before-and-after study in the same patient.

## 4 Potential mechanisms by which PDD might increase the risk of PCOS

### 4.1 PDD leads to low-grade inflammation and oxidative damage

**Figure 2:**
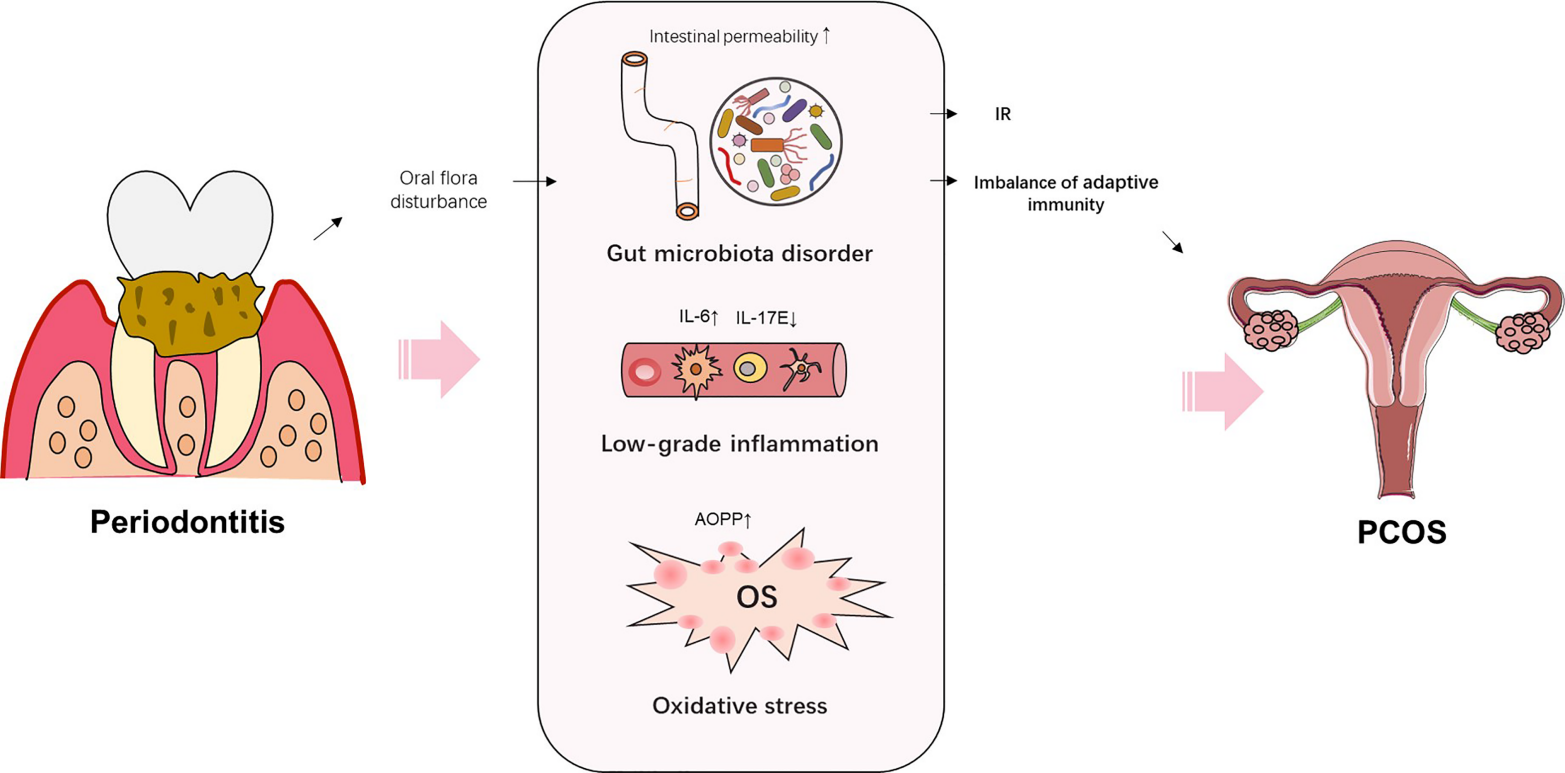
Schematic representation of the potential mechanisms by which periodontitis increases the risk of PCOS.

PDD is characterized by chronic inflammation induced by the subgingival biofilm. Various studies have demonstrated that multiple proinflammatory cytokines and reactive oxygen species (ROS) are involved in the systemic effects of PDD on systemic diseases, such as DM, MS, and obesity (88). PDD increases the risk of PCOS by mediating the anti-inflammatory and proinflammatory pathways. Higher concentrations of IL-6 in GCF, saliva, and serum were found in PCOS women with gingivitis, compared with PCOS women with healthy periodontium (16). Another study found that the expression of anti-inflammatory cytokine IL-17E was decreased in PCOS women with gingivitis, compared with PCOS women with healthy periodontium (24). In addition, PDD increases the oxidative damage and induces OS. Dharuman et al. found higher levels of advanced oxidation protein products (AOPP, a marker of oxidative damage) in the serum and saliva of PCOS women accompanied with PDD, when compared with that of PCOS women with good periodontal heath (28) (**Figure 2**). Hence, PDD might function as a risk factor for PCOS through promoting inflammation and oxidative damage.

### 4.2 PDD promotes IR

It has been confirmed that IR is accompanied by chronic and low-grade inflammation (89). Several proinflammatory cytokines are induced by PDD, including IL-1, IL-6, and TNF-α, and play critical roles in the progression of IR. Periodontitis exacerbates IR (90) and impairs the host immune response (49), which finally contributes to the development of PCOS. Subgingival flora dysbiosis might function as an aggravating factor for IR by increasing the proteobacteria levels (51). In addition, high abundance of *P.g*, a major pathogen in periodontitis, can exacerbate IR (84). Enhanced IR was observed in high-fat-fed rats after injection with *P.g*, which reveals a positive relationship between *P.g* abundance and IR and bone loss (49). The outer membrane vesicles from *P.g* decreased the insulin sensitivity through delivering gingipains to the liver (91). Another study indicated that *P.g* promoted IR in high-fat-diet-fed mice by increasing plasma levels of branched-chain amino acids (BCAAs) (92). IR alters the oral microflora composition and *vice versa (* 84). In addition, PDD impaired the β-cell function by downregulating the IL-12 levels and contributed to the development of DM (93, 94). The above findings suggest the possible harmful effects of periodontal pathogens on IR.

### 4.3 PDD induces dysbiosis of intestinal flora

Intestinal flora dysbiosis has an impact on IR and PCOS occurrence by regulating IL-22 (95). Mice transplanted with fecal microbiota from PCOS women were characterized by ovarian dysfunction, infertility, and IR, which was similar to PCOS symptoms (95). Elevated abundance of *Bacteroides vulgatus* was identified in gut microbiota of PCOS women (95). In addition, the gut microbiota disturbance in PCOS participates in the alteration of host metabolism. The levels of glycodeoxycholic acid and tauroursodeoxycholic acid were decreased in the stool and serum of PCOS women (95). Another study demonstrated a low concentration of 5-hydroxyindoleacetic acid (5-HIAA) in the serum of PCOS women (96). Disturbance of the salivary microbiota in PDD leads to dysbiosis of the gut microbiota (97). The salivary microbes in periodontitis persists in the intestine and induces intestinal microbiota dysbiosis (97). The altered composition of the gut microbiota was characterized by the enrichment of *Porphyromonadaceae* and *F.n* in mice with severe periodontitis. In addition, transplantation of PDD-related salivary microbes into the colon could initiate the inflammation in the colon by upregulating the levels of proinflammatory cytokines and chemokines in mice (97). Administration of *P.g* for mice altered the composition and function of the intestinal microbiota and even increased the intestinal permeability (90). Both tryptophan and choline metabolisms play important roles in the *P.g*-induced MS (90). In addition, the alterations of serum metabolome markers (including 5-HIAA, indole-3-acetaldehyde, P-salicylic acid, and phosphatidylcholine) were observed in mice with *P.g* administration, which is closely associated with gut microbiota. Hence, periodontitis might render individuals more susceptible to PCOS through altering the intestinal microbiota and host metabolism and increasing intestinal permeability. However, further studies are needed to clarify the effects of PDD-induced oral microbiota disorder on PCOS.

### 4.4 Periodontal therapy improves PCOS

It is essential to investigate the impact of periodontal therapy on PCOS. Deepti et al. (33) evaluated the alteration of anthropometric parameters and metabolic and periodontal parameters in periodontitis patients with PCOS after periodontal therapy for 6 months. A combination of oral hygiene instructions (OHI) or scaling and root planing (SRP) with myo-inositol (MI) significantly decreased the serum level of hsCRP in PCOS women with periodontitis. Interestingly, PDD patients with PCOS receiving SRP and MI intervention exhibited the improved body mass index (BMI) compared with those receiving OHI and MI intervention. No statistical difference was observed in serum LH/FSH, testosterone, prolactin, HOMR, and lipid profiles in both groups at 6-month follow-up. In addition, the BMI and the modified Ferriman–Gallwey score (MFG), which assess hair growth, remained high in the two groups at 6-month follow-up when compared with healthy controls. The above evidence indicates that periodontal therapy alleviates PCOS by reducing the low-grade inflammation. Hence, good oral hygiene practices and regular oral health examination are recommended for women with PCOS.

## 5 Common risk factors for PCOS and PDD

### 5.1 Genetic/epigenetic predisposition

There is little evidence reporting the impact of genetic or epigenetic modification in both PDD and PCOS. Peroxisome-proliferator-activated receptor gamma (PPAR-γ), primarily expressed in adipose tissues, exerts an influence of insulin sensitivity through regulating glucolipid metabolism (98). Both PCOS and PDD have gene polymorphisms of PPAR-γ (88, 99), which might function as a cross-link between PDD and PCOS. In addition, epigenetic alteration occurs in both diseases to regulate transcriptional events without changing the DNA sequence. Decreased methylation levels of TNF-α, COX2, IFN-γ, and immune-related genes were found in PDD (100). There were increased methylation levels of PPARG, PPARGC1A, and CYP19A1 in adipose tissues, peripheral blood, and ovarian tissues, respectively, which regulate the ovarian functions in PCOS (101, 102). Future researchers should focus on the mechanism of epigenetic alterations for the association between PCOS and PDD.

### 5.2 Low socioeconomic status

Environmental factors have significant impact on the development of PCOS and PDD. Several studies have confirmed that women with low socioeconomic status have higher incidence of PCOS (103). Meanwhile, individuals with low socioeconomic status have a higher risk of moderate-to-severe periodontitis (104, 105). Individuals with lower socioeconomic status are prone to have adverse health behaviors, including smoking, sedentary lifestyles, poor oral hygiene, and poor nutritional diet (106). Food habits with high consumption of fatty and salty foods increase risk of PCOS and PDD (107, 108). Hence, women with PCOS or PDD should pay more attention to lifestyle modification, including quitting smoking and keeping healthy with exercise and a nutritious diet. More research is needed on the effect of lifestyle intervention on patients from a lower socioeconomic stratum, having either PCOS or PDD.

## 6 Conclusion

Multiple lines of evidence confirmed the bidirectional relationships between PCOS and PDD. Endocrine disorders, low-grade inflammation, immune imbalance, and OS in PCOS deteriorate the periodontal microenvironment, while PDD accelerates the development of low-grade inflammation, OS, and IR and increases the risk of PCOS. However, only a few intervention studies revealed the mechanisms underlying the causal relation between PCOS and PDD. In addition, both PCOS and PDD share common risk factors including genetic or epigenetic predisposition and low socioeconomic status. Further longitudinal studies are needed to elucidate the shared pathophysiology between PCOS and PDD.

## Author contributions

YD, JX, DW, and JT conceived the study question, and all authors participated in the study design. JX created the first draft of the manuscript. HX, PZ, ZZ, and WF made substantial contributions to drafting the article. YD revised the manuscript. All authors contributed to the article and approved the submitted version.
